# Consultation of parents and healthcare professionals in end-of-life decision-making for neonates and infants: a population-level mortality follow-back physician survey

**DOI:** 10.1186/s12887-022-03653-z

**Published:** 2022-10-14

**Authors:** Laure Dombrecht, Filip Cools, Joachim Cohen, Luc Deliens, Linde Goossens, Gunnar Naulaers, Kim Beernaert, Kenneth Chambaere

**Affiliations:** 1 End-of-Life Care Research Group, Ghent University & Vrije Universiteit Brussel (VUB), Corneel Heymanslaan 10, K3, 6th floor, room 011, 9000 Ghent, Belgium; 2 Department of Public Health and Primary Care, Ghent University, Ghent, Belgium; 3 Department of Neonatology, Universitair Ziekenhuis Brussel, Vrije Universiteit Brussel, Jette, Belgium; 4 Department of Neonatology, Ghent University Hospital, Ghent, Belgium; 5 Department of Development and Regeneration, KU Leuven, Leuven, Belgium

**Keywords:** Infant, newborn, Palliative Care, End-of-life decision, parental consultation

## Abstract

**Background:**

End-of-life decisions with potential life-shortening effect in neonates and infants are common. We aimed to evaluate how often and in what manner neonatologists consult with parents and other healthcare providers in these cases, and whether consultation is dependent on the type of end-of-life decision made.

**Methods:**

Based on all deaths under the age of one that occurred between September 2016 and December 2017 in Flanders, Belgium, a nationwide mortality follow-back survey was performed. The survey asked about different types of end-of-life decisions, and whether and why parents and/or other healthcare providers had or had not been consulted.

**Results:**

Response rate was 83% of the total population. End-of-life decisions in neonates and infants were consulted both with parents (92%) and other healthcare providers (90%), and agreement was reached between parents and healthcare providers in most cases (96%). When medication with an explicit life-shortening intent was administered parents were always consulted prior to the decision; however when medication without explicit life-shortening intention was administered parents were not consulted in 25% of the cases.

**Conclusions:**

Shared decision-making between parents and physicians in case of neonatal or infant end-of-life decision-making is the norm in daily practice. All cases without parental consultation concerned non-treatment decisions or comfort medication without explicit life-shortening intention where physicians deemed the medical situation clear and unambiguous. However, we recommend to at least inform parents of medical options, and to explore other possibilities to engage parents in reaching a shared decision. Physicians consult other healthcare providers before making an end-of-life decision in most cases.

**Supplementary Information:**

The online version contains supplementary material available at 10.1186/s12887-022-03653-z.

## Background

In severely ill neonates and infants end-of-life decisions are often made [1]. These decisions to limit intensive care or administer medication with a potential life-shortening effect are generally made in the best interests of the child, balancing benefits and burdens of medical interventions with quality of life and possibly imminent death [2]. As neonates and infants can neither define their own best interest nor participate in the decision-making process, surrogate decision-makers should be consulted [3]. International guidelines and studies suggest that these decisions should be shared between parents and involved healthcare professionals [4–7].

Where end-of-life decisions (ELDs) concern infants unable to voice their own wishes, consulting the parents is required [8, 9]. However, a recent review of the literature indicated that evidence regarding the involvement of parents in decision-making for neonates and infants is minimal [10]. Whether or not parents are consulted depends on a variety of factors, including but not limited to the decision-making culture of the country in which the decision is made, or policy of the hospital ward [9]. Additionally, some healthcare professionals might want to protect parents from the possibly negative psychological consequences of deciding if and when their child is allowed to die [11]. Therefore, parents are commonly involved in decision-making yet the final decision is often taken by the medical team [12]. The minimal available evidence on parental involvement in decision-making for a child does not consider it on a population level independent of the hospital ward in which they were treated, such as level 3 intensive care wards, peripheral hospital ward or even at home, leading to a bias in representation. The only available population-level data is outdated: in the early 2000s in Flanders and the Netherlands, 84-97% of all neonatal end-of-life decisions were discussed with parents and in 26-29% the decision was made at the explicit request of the parents [13, 14].

Similarly, with guidelines or laws in various countries stating that healthcare professionals other than the main physician responsible for the care of the infant should be consulted during the end-of-life decision-making process [9, 15, 16], available evidence about their actual involvement is scarce and outdated. In studies similar to this one in Flanders and the Netherlands in the early 2000s, 89-97% of ELDs were discussed with other healthcare personnel. The majority of these discussions were with colleague-physicians (100% in the Netherlands, 90% in Flanders), and in 27-35% of all discussed cases a nurse or other healthcare professional was consulted [13, 14].

The aim of this study is to evaluate on a population level how often and in what manner parents and other healthcare professionals are consulted in end-of-life decision-making for seriously ill neonates and infants, and to what extent this consultation is affected by the type of end-of-life decision.

## Methods

### Design, setting and participants

We conducted a population-level mortality follow-back survey, sending questionnaires to all physicians who declared the death of an infant under the age of one year residing in Flanders between September 2016 and December 2017. More information on this method can be found in previously published papers [1, 17, 18]. STROBE guidelines for reporting cross-sectional research were used (see Additional file 1).

In Belgium, withholding or withdrawing life-sustaining treatment deemed futile is considered legal. Administering medication with an explicit life-shortening intention in neonates is not considered legal, as they fall outside of the law on euthanasia for adults and capable minors [19].

### Data collection

To ensure reliability and avoid socially desirable answers, a robust method was implemented using a trusted third party as intermediary to ensure anonymity [17]. The Total Design Method was followed, including a maximum of three follow-up postal mailings [20]. Only cases where an end-of-life decision was made were considered in this paper.

### Questionnaire and variables

The questionnaire was mainly based on the validated questionnaire used in the study on end-of-life decisions in neonates and infants in Flanders in 1999-2000 [21], and aimed at identifying different types of end-of-life decisions: withholding or withdrawing potentially life-prolonging treatment, administering medication with hastening death taken into account or co-intended (the potentially life-shortening effect is not the main goal but partly intended), or administering medication with an explicit intention of hastening death. In all ELD-cases, physicians were asked if they had discussed the ELD with the parents, and if so, if the ELD was made based on an explicit request from the parents, if parents and healthcare professionals agreed on the decision made, and whether the physician thought the parents were capable of assessing the medical situation of the infant and making an adequate decision. If no discussion with parents took place the reason was asked. Physicians were also asked whether the ELD was discussed with other healthcare professionals either individually or during a team meeting, and whether the decision was discussed with an ethics committee. No definition of “a consultation” was provided to the physicians in the survey.

We obtained the following demographic and clinical patient data from the death certificates: age at death, gestational age at birth, sex, place of death, and cause of death. A deterministic linkage procedure was used to link data from the death certificate with questionnaire data. Small cells analysis was used to ensure that the linked database would prevent reidentification.

Cause of death was categorized based on a self-developed, clinically relevant categorization aimed at achieving homogenous groups with a similar cause of death without revealing detailed information regarding all individual cases. Four physicians, currently working in neonatal and prenatal care, evaluated this categorization (see Table 1 for a description) in terms of completeness to classify all possible causes of death and clarity of descriptions. Afterwards, all cases were sorted into one of these seven categories based on the underlying cause of death, denoted by ICD-10 codes, on the death certificate. This was done by a neonatologist (FC) and a researcher with experience in neonatal end-of-life care research (LDm). ICD-10 codes of other associated causes of death were taken into account when main cause of death was inconclusive. Categories are mutually exclusive.

**Table 1:** Cause of death categories in neonatology

The following cause of death categories were identified: - Prematurity and related disorders: Death due to a direct cause of prematurity, immaturity or disorders related to prematurity. For example, necrotizing enterocolitis, intraventricular hemorrhage, respiratory distress syndrome, or death due to (extremely) low birth weight or low gestational age. - Congenital anomalies - singular: Death due to a single congenital anomaly with a defect in one organ or organ system. For example, a congenital malformation of the heart or a spina bifida. - Congenital anomalies - multiple or systemic disorders: Death due to the presence of multiple congenital anomalies in different organ systems, or due to a disorder that affects multiple organ systems. For example, chromosomal disorders, multiple congenital malformations diagnosed in one infant, or an inborn error of metabolism. - Complications of pregnancy with repercussions on foetal growth or development: Infant died due to complications of pregnancy that had an influence on the growth or the health of the baby prenatally. For example, a cytomegalovirus infection with congenital infection of the foetus, or pre-eclampsia with severe intrauterine growth restriction. - Acute complications of pregnancy and/or birth in a previously healthy foetus. For example, a placental abruption or birth trauma causing oxygen deprivation. - Disorders acquired after birth: Death due to a non-congenital disorder, acquired after birth of a previously healthy baby. For example, infectious diseases resulting in multiple organ failure. - Other: Cause of death was sudden, without previous diagnoses. Examples are sudden infant death syndrome, accidents or trauma.	

### Statistical analysis

Percentages instead of absolute numbers are presented to avoid reidentification of infants, parents, physicians and hospitals due to small cells (General Data Protection Regulation (GDPR) restrictions). Detailed information can be obtained upon request. Percentages were calculated for demographic and clinical characteristics of all infants whose death was preceded by an ELD. Percentages of ELD cases discussed with parents and healthcare professionals were calculated. Chi-square tests and two-tailed Fisher’s exact tests were used to compare characteristics of consultation with parents and healthcare professionals (explicit request from parents, agreement on the ELD, capability of parents to assess the situation, discussion with healthcare professionals individually or in team, consultation of an ethics committee) depending on the type of ELD (withholding treatment, withdrawing treatment, administration of medication with hastening death taken into account or co-intended, and administering medication with an explicit intention to hasten death). Percentages were calculated for clinical characteristics of infants where the ELD was not discussed with parents.

## Results

We received completed questionnaires for 83% of the total population of infants who died between September 1^st^ 2016 and December 31^st^ 2017. In 61.1% of cases, death was preceded by an ELD (population of this paper).

Most deaths took place in the neonatal intensive care unit (64%) (Table 2); 49% of infants died in the first week of life, 31% had a gestational age of less than 26 weeks and 37% had a gestational age of more than 36 weeks while 40.7% of all infants whose death was preceded by an ELD were diagnosed with congenital anomalies. The main causes of death (Table 1) were prematurity (20%), singular (20%) or multiple congenital anomalies (17%) or complications of the pregnancy with repercussions for the foetus (19%).

**Table 2:** demographic characteristics of all infants where death was preceded by an ELD

	%
**Sex**	
Male	61.4
Female	38.6
**Place of death**	
NICU	63.6
Other	36.4
**Age at death**	
Early neonatal death (<7 days)	49.3
Late neonatal death (7-27 days)	22.9
Post neonatal death (>27 days)	27.9
**Gestational age at birth**	
< 26 weeks	31.1
26-28 weeks	15.2
29-31 weeks	6.1
32-36 weeks	10.6
≥ 37 weeks	37.1
**Congenital anomalies**	
Yes (single or multiple)	40.7
No	59.3
**Severity of congenital anomalies**^**a**^	
Very serious	59.6
Serious	24.6
Moderate/mild^b^	15.8
**Main cause of death**	
Prematurity and related disorders	20.0
Congenital anomalies singular	20.0
Congenital anomalies multiple	17.1
Complications of the pregnancy with repercussions for the foetus	19.3
Acute complications of the pregnancy and/or birth in a healthy foetus	13.6
Disorders acquired after birth + other disorders^b^	10.0

Missing values: 6% missings in gestational age. Percentages calculated without those missing values

^a^Only answered when congenital anomalies were indicated

^b^Categories ‘moderate’ and ‘mild’; and ‘disorders acquired after birth’ and ‘other disorders’ were aggregated to protect the identity of the involved infants

In 92% of all ELD cases, the physician consulted parents prior to the ELD (Table 3). When medication was administered with an explicit intent to hasten death (100%), or when treatment was withheld (92%) or withdrawn (96%), parents were significantly more often consulted prior to the decision than when medication was administered with hastening death taken into account or co-intended (74%, p=0.002). In 28% of all consultations, the ELD was made based on an explicit request from the parents. When consultation took place, agreement about the ELD was present in 96% of cases. In 92% of all consultations, physicians indicated that parents were capable of assessing the medical situation and making an adequate decision (Table 3). The ELD was not discussed with parents in 8% of all cases, 36% of which concerned non-treatment decisions and 64% administration of medication with hastening death taken into account (not in table).

**Table 3:** consultation of parents in neonatal end-of-life decision-making

	All ELDs	Non-treatment decisions		Medication administration		*p*-value^a^
		Withholding treatment (*n*= 19% of all cases)	Withdrawing treatment (*n*= 41% of all cases)	Medication with hastening death taken into account or co-intended (*n*= 22% of all cases)	Medication with an explicit intention to hasten death (*n*= 17% of all cases)	
Consultation of physician with the parents?						0.002
Yes	92%	92%	96%	74%	100%	
No	8%	8%	4%	26%	0%	
**If parents were consulted** (n= 92% of cases):						
Was the ELD based on an explicit request from the parents?						0.076
Yes	28%	33%	22%	15%	46%	
No	72%	67%	78%	85%	54%	
Was there agreement about the ELD?						0.645
Yes, with both parents	96%	92%	96%	100%	96%	
Yes, with the mother	0%	0%	0%	0%	0%	
No or only with the father^b^	4%	8%	4%	0%	4%	
Were the parents capable of assessing the medical situation of the infant and making an adequate decision?						0.617
Yes	92%	87%	92%	95%	92%	
No, not at all or not fully capable^c^	8%	13%	8%	5%	8%	

Missing values: 6% missing values in consultation of parents, 2% missing values in explicit request of parents, 2% missing values in capability of assessing the medical situation. Percentages were calculated without these missing cases

^a^Pearson chi-square test

^b^Categories 'Yes, only with the father' and 'no' were aggregated to protect the identity of the involved infants

^c^No, not fully capable' and 'No, not at all capable' were aggregated to protect the identity of the involved infants

When the ELD was not discussed with parents, 89% of physicians indicated that no consultation was needed because the medical situation was clear (Table 4). In 64% of no-consultation-cases, no intention to shorten life was indicated. In 73% the physician estimated that the decision did not cause any shortening of life. In 80% of the reported ELD-cases without consultation with parents, the reason for the ELD was indicated as ‘no real chance of survival’ (Table 4).

**Table 4:** characteristics of cases where the end-of-life decision was not discussed with parents

	***n***=8% of all cases
**Reasons for not consulting parents**	
Not needed, medical situation was clear	89%
Other	11%
**Life-shortening intention of the physician**	
No intention to shorten life	64%
Co-intention to shorten life	9%
Explicit intention to shorten life	27%
**Estimated time by which life was shortened**	
> 4 weeks	9%
1-4 weeks	0%
1-7 days	0%
< 24 hours	18%
No shortening of life	73%
**Reason for the ELD**	
No real chance of survival	80%
No hope of a bearable future	0%
Other	20%

Missing values: 18% missing values in reasons for not consulting parents, 9% missing value in reason for the ELD. Percentages were calculated without these missing cases

In 43% of all ELD-cases, the physician consulted other physicians and/or healthcare professionals individually and in 56% of cases the decision was discussed during an open team meeting (Table 4, categories not mutually exclusive). In 10% of cases no other clinicians and/or healthcare professionals were consulted. No significant differences were found in whether a consultation with other clinicians/healthcare professionals took place depending on the type of ELD made. Certifying physicians most often consulted a neonatologist (71%). Gynaecologists were significantly more often involved in ELD discussions when withholding treatment was decided (*p*<0.001). An ethics committee was consulted in 2% of all ELD cases, in all of these cases the ELD was administration of medication with an explicit intention to hasten death (Table 5). In 3.6% of all cases no parents or other healthcare professionals were consulted (not in table).

**Table 5:** consultation of other healthcare professionals in neonatal end-of-life decision-making

	All ELDs	Non-treatment decisions		Medication administration		*p*-value
		Withholding treatment (*n*= 19% of all cases)	Withdrawing treatment (*n*= 41% of all cases)	Medication with hastening death taken into account or co-intended (*n*= 22% of all cases)	Medication with an explicit intention to hasten death (*n*= 17% of all cases)	
Consultation with other physicians/healthcare professionals (multiple answers possible)						
Yes, with individual colleagues	43%	50%	44%	37%	38%	0.741
Yes, during an open team meeting	56%	39%	57%	59%	67%	0.212
No	10%	12%	9%	19%	0%	0.189
If consulted with other professionals, who? (multiple answers possible)						
Neonatologist	71%	74%	68%	73%	91%	0.241
Nurse	42%	35%	40%	58%	48%	0.144
Pediatrician	30%	26%	40%	23%	23%	0.388
Other physician	27%	13%	38%	27%	23%	0.165
Gynecologist	20%	52%	11%	12%	22%	<0.001
Family other than the parents	11%	9%	11%	12%	13%	0.980
Others^a^	3%	0%	6%	4%	0%	0.444
Was an ethics committee consulted?						0.003
Yes, before the ELD	2%	0%	0%	0%	13%	
Yes, after the ELD	0%	0%	0%	0%	0%	
No	98%	100%	100%	100%	87%	

Missing values: 6% missing values in consultation with other physicians/healthcare professionals, 12% missing values in who was consulted and if an ethics committee was consulted. Percentages were calculated without these missing cases

^a^others include: intercultural mediator, psychologist, second opinion of other hospital

## Discussion

This population-level mortality follow-back survey indicated that end-of-life decisions in neonates and infants who died before they reached the age of one were consulted on both with parents (92%) and with other health care professionals (90%), and agreement was reached between the parents and the professionals in most cases (96%). When medication with an explicit life-shortening intent was administered parents were always consulted prior to the decision, and in 46% of these cases parents explicitly requested the decision. However, when medication without explicit life-shortening intention was administered parents were not consulted in 25% of cases. The main reason indicated by the treating physicians for not including parents in the decision-making process was because the medical situation was clear. Although the end-of-life decision was almost always discussed with colleagues, an ethical committee was only rarely consulted (2%) and all of these cases concerned decisions to administer medication with explicit life-shortening intent.

### Strengths and limitations

Despite the sensitive topic, we achieved a very high response rate (83%) by using a robust population design with a rigorous follow-up procedure [17]. This makes valid conclusions for the entire population of deceased infants under the age of one irrespective of their diagnosis or care setting highly likely. By ensuring anonymity, socially desirable answers were minimised.

Because questionnaires were filled out three months after death, recall bias could not be excluded. However, the only available registration method of deceased infants on population level is a death certificate, making it the best method to study medical situations preceding death [17]. Additionally, because the death of an infant or minor is a rare and intense event for the professionals involved, we expect recall bias to have played a smaller role than in studies of ELDs in adults^14^. For the purpose of this study, we deemed the physician’s perspective as the most important in describing the medical situation preceding death; however, viewpoints on whether a decision is really shared can differ between parents and physicians. The perspective of parents or other healthcare professionals would have been beneficial to provide a full overview of who is involved in end-of-life decision-making, however the strong anonymity guaranteed by our survey method made identifying them impossible.

### General discussion

Compared with the previous Flemish study on ELDs in neonates and infants in 1999-2000 [13], consultation with parents has slightly increased (92% versus 84%), indicating that international recommendations to move towards shared decision-making in neonatal end-of-life decision-making [4, 6, 7, 22] are being adopted by Flemish neonatologists. Consultation with parents was lowest in cases of administration of pain and/or symptom relief with a potentially life-shortening effect (74%). Possibly, this was not considered as an ELD by the professionals involved but as part of good palliative care and therefore not always explicitly discussed with parents. When possible, we recommend that in these cases parents are at least informed of the different medical options and consequences of the decisions beforehand.

In 72% of all end-of-life decisions where parents were consulted, no explicit request for the ELD is made by the parents. We hypothesize that, as healthcare professionals are the first to look for information leading to a diagnosis, treatment options are discussed first between them. When bad news conversations with parents take place shortly afterwards, these treatment options will then be offered, and a shared decision between physicians and parents is made based on recommendations made by the medical team [11, 12]. In contrast however, explicit requests from parents are far more prevalent in decisions to explicitly hasten death by means of medication (46%) than in other types of ELDs. We hypothesize that these cases predominantly arise during an unexpectedly long dying process, where questions of comfort for the child might cause parents to ask for higher doses of pain and/or symptom relief medication, thereby hastening the end of life. In these cases, we highly recommend acknowledging parents making such requests, and continuing to include them in deciding whether or not such decision is warranted. Interestingly, while only 2% of all cases were taken to an ethics committee, they all concerned decisions to administer medication with an explicit life-shortening intention, which is unlawful within the Belgian legal framework. In these rare cases, ample time was provided to consult the committee. It is possible that in these cases of unclear legislation, confirmation from an external body was warranted to resolve disagreement amongst the medical team.

Only in a minority of ELD cases were parents not consulted (8%). In most of those, physicians indicated that the medical situation was clear and unambiguous (89%) and consultation was therefore not needed. This is further corroborated by the fact that in 45% of cases without parental consultation (and 3.6% of all ELD cases), no other healthcare professionals were consulted. All of these cases concern either non-treatment decisions or medication to alleviate pain and/or symptoms without explicit life-shortening intention. In these clear cases, we hypothesize that consultation with parents would not change the outcome for the child, as the prognosis was inevitable, and parents were thereby spared the burden of having to make a decision [11, 23]. In 4% of ELDs parents were consulted, but no agreement with the medical team could be reached regarding the ELD. In all those cases, the physician indicated that the parents were not fully capable of understanding the medical situation. This could indicate some form of paternalism [24], where physicians decide what is best for the child without parental consultation. However, even when physicians deem parents incapable of understanding the diagnosis and prognosis of their child, shared decision-making is generally considered the norm. In such cases we recommend to explore all possibilities to engage with parents in reaching a shared decision.

During the end-of-life decision-making process other healthcare professionals are consulted in a majority of cases (90%), regardless of the type of decision being made. This is consistent with most guidelines on end-of-life decision-making, where gathering at least one second opinion is recommended [9, 15, 16]. In contrast, consultating an ethics committee is rare (2% of cases) indicating that physicians treating seriously ill infants under the age of one acknowledge the importance of multidisciplinary consultation, but prefer to consult specialists in the specific area of expertise than an external body such as an ethics committee. Additionally, only discrete ethical questions are presented to an ethics committee; where they have provided opinons on similar situations in the past such questions are not repeated. Our results show that physicians mainly consult other neonatologists (71%), suggesting that regular team meetings discussing all patients on a specific neonatal ward are probably the main source of second opinions. Additionally, our data suggest that physicians appreciate the opinion of specialist-physicians more than those of nurses in the department, as nurses are consulted in less than half of all cases. This indicates that including nurses in decision-making continues to be challenging, for example because nurses working in shifts are often unable to be present at team meetings, or because they feel unable to voice their opinion [25]. However, nurses can provide valuable information regarding both parental values and wishes and the medical condition of the infant, as they spend many hours providing daily care for the infants [26]. We thus highly suggest including nurses in decision-making. Additionally, the COVID-19 pandemic caused an increase in digital meetings, making the involvement of nurses who would otherwise not be available easier. Including a perinatal palliative care team might be beneficial in this regard, as these multidisciplinary teams, including nurses trained specifically in covering the palliative care needs of all involved [27], have ample experience in both providing care at the end of life and in including all team members in decision-making.

## Conclusion

This study shows that physicians consult with parents and colleagues prior to making end-of-life decisions in almost all cases of neonates and infants who died before the age of one, indicating that shared decision-making is the norm in daily practice. All cases without parental consultation involved non-treatment decisions or comfort medication without explicit life-shortening intention, where physicians deemed the medical situation clear and unambiguous. However, we recommend that those concerend at least inform parents of the medical options and explore other possibilities of engaging them in reaching a shared decision. Second opinions are most often sought of fellow neonatologists while nurse involvement is low; however, nurses can provide valuable information regarding the medical status of the child and the values and wishes of the parents and thus their involvement in decision-making could be encouraged.

## Supplementary Information

**Additional file 1.**

## Data Availability

Questionnaires and detailed research protocols (in Dutch) used in the current study are available upon written request to the corresponding author (Laure.Dombrecht@UGent.be).
